# An Attempt to Replace Pure Citric Acid with Natural Lemon Juice during Potato Starch Esterification

**DOI:** 10.3390/molecules29112687

**Published:** 2024-06-06

**Authors:** Ewa Tomaszewska-Ciosk, Ewa Zdybel, Małgorzata Kapelko-Żeberska, Bartosz Raszewski, Krzysztof Buksa, Agnieszka Maj, Tomasz Zięba, Artur Gryszkin

**Affiliations:** 1 The Faculty of Food Science, Wroclaw University of Environmental and Life Sciences, Chełmońskiego 37/41, 51-630 Wroclaw, Poland; ewa.tomaszewska-ciosk@upwr.edu.pl (E.T.-C.); ewa.zdybel@upwr.edu.pl (E.Z.); bartosz.raszewski@upwr.edu.pl (B.R.); tomasz.zieba@upwr.edu.pl (T.Z.); artur.gryszkin@upwr.edu.pl (A.G.); 2 Department of Carbohydrate Technology and Cereal Processing, University of Agriculture in Krakow, Balicka 122, 30-149 Krakow, Poland; krzysiek_b@onet.eu; 3 Institute of Sport, Tourism and Nutrition, Faculty of Biological Sciences, University of Zielona Góra, Licealna 9, 65-417 Zielona Góra, Poland; amaj@uz.zgora.pl

**Keywords:** potato starch, lemon juice, esterification, resistant starch

## Abstract

The application of chemical operations in food processing, in which pure chemical compounds are used to modify food ingredients, often raises social concerns. One of the most frequently modified dietary substances is starch, e.g., E1401–E1404, E1412–E1414, E1420, E1422, E1440, E1442, and E1450–E1452. An alternative solution to chemical treatments seems to be the use of raw materials naturally containing substrates applied for starch modification. Heating starch with a lemon juice concentrate can be considered a novel and effective method for producing starch citrate, which is part of the so-called “green chemistry”. The modified preparations obtained as a result of potato starch esterification with natural lemon juice had a comparable degree of esterification to that of the esters produced with pure citric acid. In addition, the use of the juice doubled their resistance to amylolytic enzymes compared to the preparations made with pure acid. Replacing citric acid with lemon juice can facilitate the esterification process, and the analyzed properties of both types of modified preparations indicate that starch esters produced with pure citric acid can be successfully replaced by those produced using natural lemon juice, which may increase the social acceptance of these modified preparations.

## 1. Introduction

**Figure 1:**
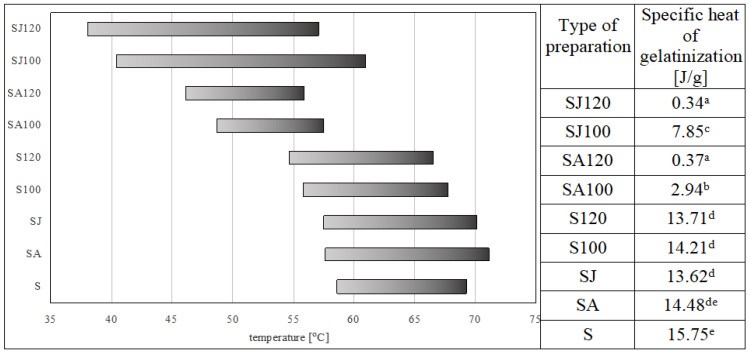
Temperature range and specific heat of gelatinization of starch citrates obtained during starch roasting with citric acid from various sources (Different letters means homogeneous groups at *p* < 0.05).

Food production and gastronomic preparation of dishes entail heat treatment. Such processes as drying, cooking, baking, frying, or roasting are conducted at temperatures over 100 °C and result in intense physical and chemical changes taking place in the processed raw materials or semi-finished products, imparting the desired sensory characteristics to the finished product. These reactions are described in detail in academic textbooks on food chemistry [1,2]. As a result of the heat supplied, proteins are denatured and new permanent cross-linking bonds are formed, triggering changes in the hardness, solubility, and water-holding capacity of the protein material. Together with lipids, partially degraded myofibrillar proteins and collagen form aggregates exhibiting properties of a viscoelastic plastic body. At elevated temperatures, lipids undergo hydrolytic changes and oxidation. Starch undergoes pasting in an aqueous environment, and when heated at high temperatures, it can undergo acid hydrolysis, thermolysis, and repolymerization, while its long-term roasting leads to the formation of formic acid and formaldehyde, acetaldehyde, and furan. The partial breakdown of proteins, lipids, and saccharides leads to the formation of flavor and aroma compounds characteristic of a given product. Chemical reactions (e.g., Maillard reactions) lead to a change in color typical of heat-treated products. Kitchen accessories and devices, such as an oven, pot, pan, or microwave oven, are reactors in which substances naturally present in raw materials undergo numerous chemical transformations. The natural origin of these substances and the habit of traditional culinary processing of food raise no concerns among consumers. However, the use of chemical substances (so-called preservatives) for food preservation or the application of chemical operations in food technology, in which pure chemical compounds are used to modify food ingredients, raises serious social objectives. One of the raw materials subjected to this type of treatment is starch. To adjust its properties to a given application, it is subjected to esterification, etherification, or oxidation reactions. Chemically modified starch preparations produced in this way can be used as food additives (E1401–E1404, E1412–E1414, E1420, E1422, E1440, E1442, E1450–E1452) [3]. An alternative solution to chemical operations deployed in food technology seems to be the use of raw materials naturally containing substrates applicable for starch modifications. In earlier works, attempts were made to produce modified starch preparations using organic acids naturally occurring in microbiological culture media [4,5]. However, these studies failed to produce starch esters with a high degree of esterification. When looking for the possibilities of efficient starch modification, one should search for a raw material containing a reaction substrate with a high affinity to starch, with a possibly high content of a reaction substitute and a low content of other substances that can be esterified. A natural raw material that meets these requirements seems to be lemon juice. It contains up to 8% citric acid [6], which has three carboxyl groups, two of which easily react with the hydroxyl groups of starch. Starch roasting with citric acid allows for obtaining starch cross-linked with citric acid (Figure 1). Starch citrates exhibit different properties depending on the degree of esterification [7], and previous investigations have indicated that they can be used as resistant starch preparations [8,9,10] or as components of biodegradable packaging materials [11].

The aim of the present study was to produce a highly substituted citrate using potato starch and lemon juice concentrate as a substrate, and to compare its properties with a preparation produced with a standard method. The second study goal was to use the lemon juice concentrate as a natural substrate for starch esterification. The properties of the modified citrate were compared with those of the traditionally produced preparation. Finally, the study identified the feasibility of producing starch citrates with this novel, alternative method.

## 2. Discussion of Results

### 2.1. Percentage of Esterification of Starch Citrates

Heating starch with citric acid or lemon juice allowed for the production of starch esters, whose percentage of esterification ranged from 2.1 to 4.86% (Table 1). The possibility of starch esterification with citric acid under such conditions was also reported by other authors [7]. The use of both pure citric acid and the acid contained in lemon juice for esterification led to a statistically significant increase in the percentage of esterification at both reaction temperatures tested in the experiment. It should be noted that the form of the reagent used in the experiment influenced the ultimate effect of starch esterification with citric acid.

A higher percentage of esterification was obtained with pure citric acid (4.86%) than with acid from the lemon juice (3.55%). The accompanying substances, probably present in the juice, e.g., simple sugars, reacted with the acid, forming water-soluble short-chain esters, which were washed out during the technological process, thus reducing the percentage of esterification. Similar results were also obtained by other authors who esterified starch with citric acid [4,5]. By esterifying starch with citric acid contained in the culture medium of *Yarrowia lipolytica* yeast, Zdybel et al. also observed the influence of the presence of other compounds on the reaction efficiency, compared to model samples prepared using pure acid solutions (analytically pure) [5]. During the experiment, the determined degree of substitution was converted into citric acid. It may, however, be expected that esters of other organic acids present in the lemon juice were also formed in the preparations made of this juice. Nevertheless, a previous study by Zdybel [5] demonstrated that, despite the presence of other organic acids in the medium, citric acid was the main acid produced, whereas the production yield of the other acids was low (e.g., alfa keto-glutaric acid < 0.5%) or null (e.g., pyruvic acid) [4,5]. The higher roasting temperature used with both substrates tested increased the percentage of esterification of the final citrates. A positive correlation between the percentage of esterification and the roasting temperature was also observed by other authors in the case of esterification with citric acid [12,13] and other acids [14].

### 2.2. Swelling Power and Water Solubility of Starch Citrates

The results of determinations of the percentage of starch citrate esterification were confirmed by the results of determinations of water-holding capacity (WHC) and swelling power. Along with the increase in the percentage of esterification, the values of WHC and the swelling power increased as well in the range of 1.45–4.08 (Table 2) and 2.12–6.35 (Table 3), respectively. Due to its chemical structure, when included in the starch structure, citric acid causes not only its esterification but also cross-linking, which significantly increases the water-holding capacity of the modified preparations [10,11]. Differences in water absorption by starch are due to the content of amylose and amylopectin in the starch granules [15]. The fraction that causes greater swelling and water absorption is amylopectin, which forms structures that allow water to be easily incorporated. This was also confirmed by Liu et al., who analyzed the effect of the chain length ratio in fractions and chain distribution in granules on their swelling [16,17]. Cross-linking with citric acid causes the expansion and greater complexity of the structure of starch chains, which can result in an increase in water-holding capacity similar to the increase described by Liu et al. [17]. Citrates made with lemon juice were characterized by lower water-holding capacity compared to the analogous preparations made with pure citric acid. This was probably due to the lower percentage of esterification achieved in these preparations. However, Hong’s research group showed that the affinity to water depended not only on the percentage of esterification, but mainly on the production technology of the modified preparations [18]. In turn, other authors have demonstrated a change in the swelling power of the starch preparation produced from starches of various botanical origins and modified with citric acid compared to the preparations modified with other acids [19,20,21]. They showed that starch esters with other acids, e.g., with lactic acid [19], caused a decrease in water absorption compared to starch citrates. It is probable that other acids (including malic acid) contained in lemon juice also formed esters with starch [6]. Likewise, regarding lactic acid esters, these esters could also probably decrease the swelling power and water-holding capacity of the modified preparations. This fact additionally explains the lower water-holding capacity values obtained in this study for the preparations obtained with lemon juice compared to those produced with pure citric acid.

### 2.3. Resistance of Starch Citrates to the Activity of Amyloglucosidase

One of the key properties of starch is its resistance to enzymatic hydrolysis. According to literature reports, the content of resistant starch in starch citrates produced via various methods and from various raw materials ranges from a few to even 100% [8,12,22,23]. Resistance of roasted native starch to amyloglucosidases ranged from 0.5 to 0.75% (Table 4) and was attributed to depolymerization reactions taking place at elevated temperatures [15]. The resistance of the modified preparations produced by starch cross-linking with citric acid oscillated in the range of 0.75–43.29% (Table 4). Replacing pure citric acid with lemon juice each time resulted in a statistically significant increase in the resistance of the modified preparations, both the non-roasted ones and those produced at high temperatures. The roasting temperature also had a significant effect on the resistance of cross-linked starch. Both the modified preparations obtained with pure acid and lemon juice exhibited a higher resistance when roasted at 120 °C than at 100 °C. Studies published so far [5,8,12,21,22,24] have confirmed the correlation between starch resistance and the dose of acid used, while in the studies by Kim and Lee, the authors showed a significant dependence of the reaction temperature on resistant starch content [25,26]. The formation of the resistant starch fraction results from a large interference in the structure of starch chains, i.e., from the incorporation of citric acid residues into these chains, which, by connecting various sites of the chains, causes their cross-linking and hinders the access of amylolytic enzymes [8,12,26,27].

When lemon juice was used for starch cross-linking, the modified preparations featured a lower percentage of esterification, but their resistance was higher compared to that of the esters obtained with pure citric acid. This was probably due to the inclusion of other substances present in the juice into the structure of starch chains, which resulted in more intensive remodeling of their structure. In addition, the resistance is determined not only by the percentage of esterification, but also by the place of substitution of acid residues. Zięba et al. [28] proved that the acetylation of starch at the 3rd and 2nd carbon atoms increased the resistance to a greater extent than the acetylation of starch at the 6th carbon atom. Among the modified preparations tested, the highest resistance (95.15%) was found for the one produced with lemon juice at the roasting temperature of 120 °C. The simultaneous use of high temperature and the inhomogeneous substrate enabled obtaining a preparation highly resistant to enzymes. A similar effect of temperature and inhomogeneous substrate was noted by Zdybel et al. [5].

### 2.4. Thermal Properties of Starch Citrates

The gelatinization temperature of native starch (without reagent addition and non-roasted) was in the range of 58.58–69.29 °C (Figure 1) and was found typical of native potato starch [24]. Reagent addition to the non-roasted samples caused an extension of the gelatinization temperature range—a decrease in the initial temperature by about 1 °C in the esters produced with the acid and with the juice and an increase in the final transition temperature by about 1 °C in the esters made with the juice and by 2 °C in those made with the acid. The increase in the temperature range in those samples was due to environmental pH drop [29]. The roasting process performed without reagent addition also causes a decrease in the initial transition temperature but does not increase the temperature range because it additionally lowers the final temperature of transition. Starch roasting at temperatures over 100 °C causes disorders in the crystalline structure of the starch granules, which may be the reason for the conversion of their structure. High temperature leads to the breaking of hydrogen bonds between starch helices, amylopectin degradation, and an increase in the amylose content of the granules, which results in lesser amylose crystallinity and may facilitate the gelatinization process by lowering its temperature and the amount of energy needed [29,30,31]. The most significant changes in gelatinization temperatures were observed in the case of preparations roasted with the reagent. All these preparations were characterized by reduced gelatinization temperatures. Preparations with lemon juice roasted at 120 °C were characterized by the lowest initial gelatinization temperature, while the lowest final gelatinization temperature was found for the preparations roasted with pure citric acid at 120 °C. The synergistic effect of low pH and roasting temperature resulted in a drastic drop in gelatinization temperatures attributed to the process of dextrinization (shortening) of starch chains taking place under these conditions, as indicated by the results of the specific heat of the tested preparations [5]. The values of this parameter were also drastically reduced in the samples in which the dextrinization process occurring during roasting was intensified by low pH, with the lowest values determined for the samples roasted at 120 °C (0.37 J/g for the starch roasted with pure citric acid and 0.34 J/g for the starch roasted with lemon juice). The samples roasted with the addition of lemon juice exhibited the widest range of gelatinization temperatures (almost two times greater than the samples obtained during starch roasting with citric acid) (Figure 1). 

The stretching of the starch phase transition peak observed for the preparations roasted with lemon juice addition was caused by numerous and very complicated reactions of all the additional substances introduced with the juice (carbohydrates, fiber, and proteins). Analyzing the values of the specific heat of phase transition of the samples roasted with the reagent, it was found that the greatest loss of transition heat was caused by roasting at 120 °C (below 0.4 J/g). Roasting starch with citric acid significantly affected the thermal stability of the modified preparations. Regardless of reagent type, all obtained preparations were characterized by significantly lower enthalpy values. This was probably due to the cross-linking of the starch chains, which made the entire starch structure more rigid and prevented these chains from moving in water. Other authors also noted a decrease in the enthalpy of starch citrates in their works. Some of them achieved such a degree of cross-linking that completely prevented gelatinization, especially at higher roasting temperatures [8,12].

### 2.5. Starch Citrate Molecular Weight Distribution

It was not possible to perform the analysis of the chain length for potato starch roasted with lemon juice at 120 °C because this preparation did not dissolve completely in DMSO. This was probably due to the highly remodeled structure of the chains (esterification and cross-linking), which is also confirmed by the high enzymatic resistance of this preparation (Table 5).

Starch consists of two fractions, differing in the size of amylose and amylopectin, which makes it difficult to interpret the results obtained due to the inhomogeneity of the chains. The homogeneity of the analyzed sample is indicated by the degree of their molar dispersity (Ð = M_ww_/M_nw_), which is the quotient of the weighted average molecular weight (M_ww_) and the number of average molecular weight (M_nw_). The analysis of the average molecular weights of heterogeneous samples is very difficult and may lead to false conclusions due to the different particle size distributions of the compared samples. In order to facilitate the interpretation of the results, the chromatograms were divided into two parts, which enabled the description of high (A) and low (B) polysaccharide fractions (Table 5).

Starch roasting at temperatures above 100 °C causes changes in its crystallinity, degradation of its chains, and its thermolysis [30]. This phenomenon caused a decrease in the M_ww_ value of fraction A for the starch roasted at 120 °C (127.6) compared to that roasted at 100 °C (164.0) and non-roasted (181.4). Analyzing the results concerning the chain length of fraction B, no such clear differences were observed in their molecular weight (Table 5). Undoubtedly, fraction B was also degraded into shorter chains, but this result was distorted by fragments of chains resulting from fraction A degradation, increasing the average M_ww_ values of fraction B [28,32]. Relatively large fragments of A chains in fraction B present together with very short chains contributed to the increased heterogeneity of this fraction and translated into an increased Ð value of these samples (SJ100—29.9; SA100—13.4 and SA120—15.5) (Table 5). When analyzing the preparations with the addition of lemon juice or citric acid subjected to roasting, an increase in the average value of the molecular weight in fraction A was also found when compared to this value in the combined fractions A + B. This was probably due to two opposing processes occurring during sample roasting. At the same time, the length of the chains shortened as a result of progressing pyrolysis, and the shorter chains were esterified and cross-linked with citric acid [8,10,12], resulting in their increased molecular weight.

## 3. Materials and Methods

### 3.1. Materials

The study materials included potato starch (with an amylose content of 23.5%) produced by PEPEES SA (Łomża, Poland production date 20 September 2022), concentrated lemon juice produced by Jowam (Myślibórz, Poland) (production date July 2022), a.p. citric acid monohydrate (pH 2.36, extract 50%) from Stanilab (Wroclaw, Poland), and a.p. sodium hydroxide from Stanilab (Poland).

### 3.2. Production of Starch Preparations

**Figure 2:**
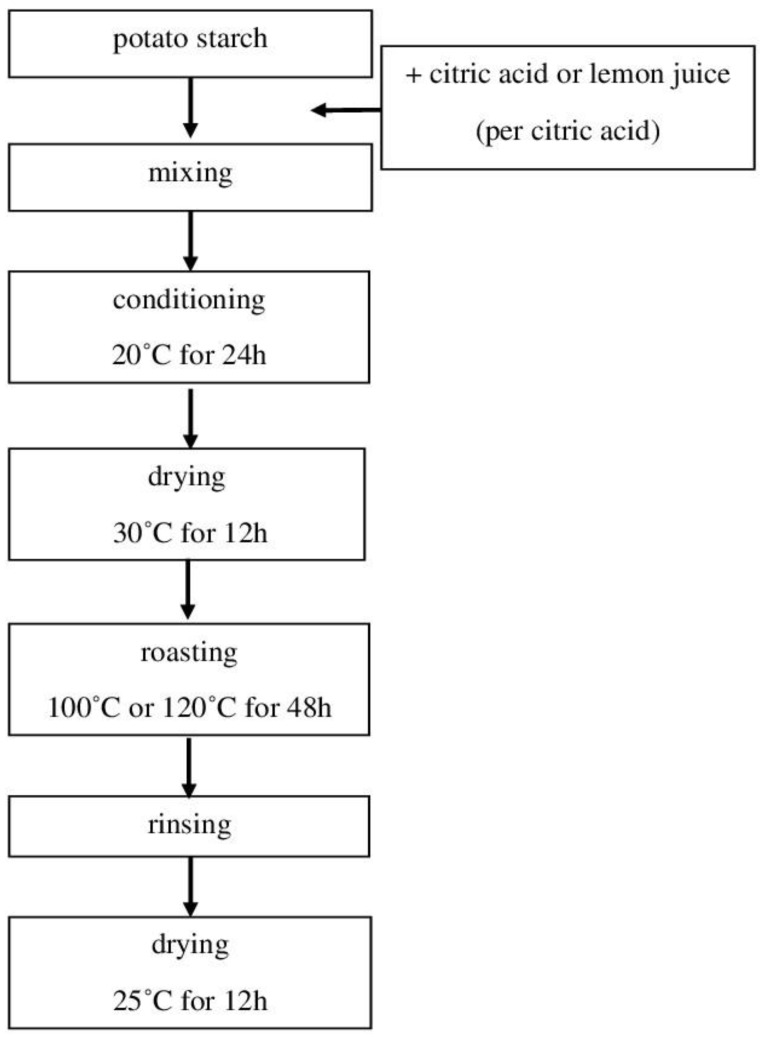
Scheme of the producing starch citrate.

Mixtures of potato starch and citric acid or lemon juice (calculated as citric acid) were prepared in the amount of 0 g or 10.0 g of citric acid per 100.0 g of starch DM. To this end, a solution of lemon juice or citric acid in water was prepared, which was modified with 10 M sodium hydroxide to pH = 3.5. The starch was mixed with the acid or juice solution, then mashed through a screen to achieve a homogenous suspension, and conditioned at room temperature for 24 h. Afterward, the samples were dried in an air dryer at 30 °C for 12 h. The dried material was roasted at 100 °C or 120 °C for 48 h. The roasted starch citrates were rinsed 30 times with distilled water to wash off excess reagent and then dried at 25 °C for 12 h (Figure 2). Non-roasted preparations served as the reference sample [8,12].

### 3.3. Determination of the Percentage of Esterification

The percentage of esterification was expressed in grams of citric acid per 100 g of starch preparations [12,33]. An amount of 2 mL of distilled water and 50 mL of 1 M potassium hydroxide solution were added to 2 g DM of the preparation, and the mixture was heated for 10 min until starch gelatinized completely. Then, it was cooled to room temperature and neutralized with 5 M acetic acid to pH = 8.5. Subsequently, 25 mL of borate buffer and approx. 0.3 g of a marker (a mixture of murexide and sodium sulfate (VI) in a ratio of 1:500) were added to the sample, and then the sample was filled up with 300 mL of distilled water. The sample prepared in this way was titrated with a 0.05 M solution of copper (II) sulfate (VI) until the pink-violet color disappeared. The assay was performed in three replications.

The percentage of esterification of the tested citrates was calculated using the following formula:

$$DE=\frac{(V\cdot 0.0961)\cdot 10}{m}$$

where *DE*—the percentage of esterification with citric acid; V—the volume of 0.05 M copper (II) sulfate (VI); and m—the mass of the tested sample converted to dry matter.

### 3.4. Determination of Water-Holding Capacity 

An amount of 200 mg of the preparation on a dry matter basis was weighed into the test tube. Then, 1.5 mL of distilled water was added, and the sample was mixed and conditioned at room temperature for 10 min. Afterward, it was centrifuged in a laboratory centrifuge MPW-55 at 5000 rpm for 10 min, and then the supernatant was decanted and the precipitate remaining in the test tube was weighed. Water-holding capacity, expressed in grams of distilled water per gram of dry preparation, was calculated according to the following formula:

$$WHC=\frac{M_{\mathrm{tp}}-M_{\mathrm{et}}-M_{w}}{M_{\mathrm{dm}}}\left[g/g\right]$$

where *WHC*—water-holding capacity; M_tp_—mass of the tube with the precipitate after centrifugation; M_et_—mass of an empty tube; M_w_—mass of a weighted portion of starch; and M_dm_—mass of dry matter in the test tube.

### 3.5. Determination of the Resistance of Starch Preparations to the Action of Amyloglucosidase

An amount of 38 g of a 0.72% suspension of the starch preparation was prepared in a conical flask and kept at the boiling temperature for 5 min [32]. After cooling the suspension, the amount of evaporated water was supplemented to a mass of 38 g, and 34 mL of acetate buffer with pH = 4.35 were added. The flask was placed in a water bath at 37 °C with an active shaker, and 4 mL of an amyloglucosidase solution (Dextrozyme DX 1.5X, NOVONESIS, Lyngby, Denmark) was added. The amount of the enzyme added was selected to ensure the complete saccharification of native starch after 2 h of hydrolysis. Every hour, 1 mL of the hydrolysate was collected into a centrifuge vessel and centrifuged at a speed of 1825× *g* for 5 min (the final result was assumed to be the value obtained when the absorbance from three consecutive measurements did not change). An amount of 10 µL of the supernatant was taken from the centrifuged sample and transferred to a cuvette by adding 1 mL of the BIOSYSTEM reagent (a kit for glucose content determination containing glucose oxidase and peroxidase, by BioSystems S.A. Barcelona Spain), then mixed and incubated for 15 min at 20 °C. Absorbance was measured using a CECIL 2000 colorimeter (Cambridge, England) at a wavelength of λ = 500 nm. Measurements were made against a blank, which was a reagent with buffer and water. The content of glucose was read from the standard curve. The resistance of the preparations was calculated from the following formula:

$$R=100-\frac{x\cdot 100}{0.396}\left[\%\right]$$

where *R*—resistance of preparations [%], x—content of glucose read from the standard curve [mg], and 0.396—the maximal amount of glucose formed from 0.36% of starch paste [mg].

### 3.6. Determination of the Swelling Power in Water

The swelling power of the preparations in water was determined as the ratio of the volumetric mean diameter measured in water and without water. The volumetric mean diameter D[4,3] of the starch preparations was determined using a Mastersizer 2000 laser particle analyzer from Malvern Instruments Ltd. (Cambridge, UK). The measurements were made in a dry mode using the Scirocco 2000 (Malvern Instruments Ltd.) attachment with sample obscurity of 1–3% and a pressure of 2 bar, and in aqueous solutions using the Hydro 2000 MU (Malvern Instruments Ltd.) attachment with sample obscurity of 10–15% and the feeding pump speed of 2000 m/min. Before the wet-mode measurement, the sample was kept in water at 30 °C for 20 min and treated with 20 Hz ultrasound for 10 s to disintegrate the agglomerates [34].

The average volumetric diameter D[4,3] measured without water and in water was determined, and the swelling power (n-fold increase in the volume of starch preparations in water) was determined according to the following formula:

$$P=\frac{{\left(D\left[4,3\right]Hydro\right)}^{3}}{{\left(D\left[4,3\right]Scirocco\right)}^{3}}$$

where *P*—swelling power; D[4,3]Scirocco—average volumetric diameter of starch granules measured without water; and D[4,3]Hydro—average volumetric diameter of starch granules measured in water.

### 3.7. Determination of the Thermal Gelatinization Characteristics of the Preparations

The thermal gelatinization characteristics were determined using a DSC 822E scanning calorimeter from Mettler Toledo (Columbus, OH, USA), using ME-5119872 aluminum dishes with a capacity of 100 µL. A sample of the tested starch preparation (10 mg) was placed in a measuring vessel, to which redistilled water was added in a ratio of 3:1 (3 parts of water per 1 part of starch). The vessels were conditioned at 25 °C for 30 min, then heated to 100 °C at a heating rate of 4 °C/min. The thermal characteristics of the tested samples, including the initial and final temperatures of the phase transition and the enthalpy of gelatinization of starch pasting, were determined based on the obtained thermographs [5].

### 3.8. Analysis of Molar Mass Distribution of Starches by Size Exclusion Chromatography

A 20 mg sample was dissolved in 6 mL of DMSO at 70 °C for 24 h or 4 mL of 0.5 M NaOH at 35 °C for 24 h using a magnetic stirrer. After dissolution, the NaOH sample was neutralized with 2 mL of 1 M HCl. In the next step, the solutions were centrifuged at 2000× *g* for 5 min, and then the supernatant was injected into the columns. An aqueous solution of 100 mM NaNO_3_ was used as the eluent. The flow rate was 0.6 mL/min, and the injection loop was 100 mL. The temperature of the columns was set at 60 °C. The molecular weight distribution of the starch was measured by refractive index (RI) detection (Knauer, Germany) and then used to calculate the following molecular parameters: average molecular weights (M_ww_, M_nw_) and molar dispersity (Ð) using Eurochrom ver. 1.13a and Clarity ver. 11.0b (DataApex, Czech Republic) software. The calibration curve was plotted with pullulan standards (Shodex Standard, Macherey—Nagel) with known molecular masses (P-5, 10, 100, 400, and 800) and glucose [35].

### 3.9. Statistical Analysis

The results were submitted to statistical analysis using Statistica v 13.0 software (Statsoft, Kraków, Poland). Homogenous groups and LSD values were denoted using Tukey’s multiple comparison test. It concerned α = 0.05, significance levels with unidirectional analysis of variation for two variables.

## 4. Conclusions

Heating starch with natural lemon juice can be considered a new and effective method for producing starch citrate, which is part of the so-called “green chemistry”. The modified preparations obtained as a result of potato starch esterification with natural lemon juice had a comparable percentage of esterification to the esters produced with pure citric acid. In addition, the use of the lemon juice doubled ester resistance to the action of amylolytic enzymes compared to the preparations made with pure acid. The water-holding capacity and the swelling power of the preparations made with natural lemon juice were lower compared to the esters made with pure citric acid. The analysis of phase transition thermograms showed that the applied heating temperature had a stronger impact on the specific heat of transition than the reactant type. A significant extension of the range of transition temperatures was also observed for the preparations roasted with lemon juice compared to those roasted with pure citric acid. The properties of the obtained preparations indicate that those produced with natural lemon juice would be weaker thickeners (lower water-holding capacity and swelling power) than those made with pure citric acid. However, due to their high resistance, they can be successfully used in products with a reduced energy value.

## Figures and Tables

**Table 1:** Percentage of esterification [%] of starch citrates obtained during starch roasting with citric acid from various sources (Different letters means homogeneous groups at *p* < 0.05).

Roasting Temperature [°C]	Substrate Source	
	Citric Acid (SA)	Lemon Juice (SJ)
non-roasted	0.08 ^a^	0.22 ^b^
100	3.62 ^d^	2.10 ^c^
120	4.86 ^e^	3.55 ^d^

**Table 2:** Water-holding capacity [g/g] of starch citrates obtained during starch roasting with citric acid from various sources (Different letters means homogeneous groups at *p* < 0.05).

Roasting Temperature [°C]	Substrate Source		
	None (S)	Citric Acid (SA)	Lemon Juice (SJ)
non-roasted	1.17 ^a^	1.16 ^a^	1.18 ^a^
100	1.14 ^a^	3.95 ^d^	1.45 ^b^
120	1.15 ^a^	4.08 ^d^	2.57 ^c^

**Table 3:** Swelling power [ ] of starch citrates obtained during starch roasting with citric acid from various sources (Different letters means homogeneous groups at *p* < 0.05).

Roasting Temperature [°C]	Substrate Source		
	None (S)	Citric Acid (SA)	Lemon Juice (SJ)
non-roasted	1.42 ^b^	1.23 ^a^	1.15 ^a^
100	1.16 ^a^	4.85 ^e^	2.12 ^c^
120	1.10 ^a^	6.35 ^f^	4.16 ^d^

**Table 4:** Resistance [%] of starch citrates obtained during starch roasting with citric acid from various sources to amyloglucosidase (Different letters means homogeneous groups at *p* < 0.05).

Roasting Temperature [°C]	Substrate Source		
	None (S)	Citric Acid (SA)	Lemon Juice (SJ)
non-roasted	0.50 ^a^	0.75 ^a^	1.66 ^a^
100	0.68 ^a^	14.37 ^b^	37.49 ^c^
120	0.75 ^a^	43.29 ^d^	95.15 ^e^

**Table 5:** Weighted average molecular weight (M_ww_) and degree of molar dispersity (Ð) of starch citrates obtained during starch roasting with citric acid from various sources (* preparation did not completely dissolve in DMSO).

Roasting Temperature [°C]		Substrate Source					
		None (S)		Citric Acid (SA)		Lemon Juice (SJ)	
		M_ww_ × 10^4^ [g/mol]	Ð	M_ww_ × 10^4^ [g/mol]	Ð	M_ww_ × 10^4^ [g/mol]	Ð
non-roasted	A + B	181.4	3.8	157.0	6.9	131.5	4.8
	A	226.2	1.7	207.8	1.7	177.9	1.6
	B	22.3	2.6	20.7	3.5	21.0	2.6
100	A + B	116.6	6.7	53.8	9.9	62.9	19.2
	A	164.0	1.5	155.8	1.5	158.2	1.6
	B	20.3	3.5	11.6	13.4	6.2	29.9
120	A + B	75.2	4.9	43.4	13.8	*	*
	A	127.6	1.4	137.7	1.4	*	*
	B	19.8	3.4	7.9	15.6	*	*

## Data Availability

Data are contained within the article.

## Abbreviations

The following preparations were obtained: S—native starch, SA—starch with pure citric acid, SJ—starch with natural lemon juice, S100—native starch roasted at 100 °C, S120—native starch roasted at 120 °C, SA100—starch with pure citric acid roasted at 100 °C, SA120—starch with pure citric acid roasted at 120 °C, SJ100—starch with natural lemon juice roasted at 100 °C, SJ120—starch with natural lemon juice roasted at 120 °C.
